# Supplementary material for: TMEM120A is a coenzyme A-binding membrane protein with structural similarities to ELOVL fatty acid elongase
Source: eLife. 2021 Aug 10;10:e71220. doi: 10.7554/eLife.71220 (PMC8376247; doi:10.7554/eLife.71220)
Supplement: Figure 2—source data 1. [file elife-71220-fig2-data1.doc]

| TMEM120A (EMD: 24230) (PDB: 7N7P) | | | | |
| --- | --- | --- | --- | --- |
| **Data collection and processing** | |  | **Refinement** | |
| Magnification  Voltage (kV) | 81,000  300 |  | Model resolution (Å)  FSC threshold | 3.24  0.143 |
| Electron exposure (e–/Å^2^) | 60 |  | Map sharpening *B* factor (Å^2^) | -142.67 |
| Defocus range (μm) | -0.9 - -2.2 |  | Model composition  Non-hydrogen atoms  Protein residues  Ligands (CoA) | 5400  626  2 |
| Pixel size (Å) | 0.844 |  | *B* factors (Å^2^)  Protein  Ligand | 51.29  44.42 |
| Symmetry imposed | C2 |  | R.m.s. deviations  Bond lengths (Å)  Bond angles (°) | 0.005  0.940 |
| Initial particle images (no.)  Final particle images (no.) | 1,838,840  701,283 |  | Validation  MolProbity score  Clashscore  Poor rotamers (%) | 1.17  3.81  0.35 |
| Map resolution (Å)  FSC threshold | 3.24  0.143 |  | Ramachandran plot  Favored (%)  Allowed (%)  Disallowed (%) | 98.37  1.63  0 |
